# Preweaning period is a critical window for rumen microbial regulation of average daily gain in Holstein heifer calves

**DOI:** 10.1186/s40104-023-00934-0

**Published:** 2023-11-02

**Authors:** Shengyang Xu, Chong Jiao, Qiyu Diao, Yan Tu

**Affiliations:** Beijing Key Laboratory for Dairy Cow Nutrition, Institute of Feed Research, Chinese Academy of Agricultural Sciences, Beijing, 100081 People’s Republic of China

**Keywords:** Average daily gain, Calves, Preweaning period, Rumen microbiota

## Abstract

**Background:**

Rumen bacterial groups can affect growth performance, such as average daily gain (ADG), feed intake, and efficiency. The study aimed to investigate the inter-relationship of rumen bacterial composition, rumen fermentation indicators, serum indicators, and growth performance of Holstein heifer calves with different ADG. Twelve calves were chosen from a trail with 60 calves and divided into higher ADG (HADG, high pre- and post-weaning ADG, *n* = 6) and lower ADG (LADG, low pre- and post-weaning ADG, *n* = 6) groups to investigate differences in bacterial composition and functions and host phenotype.

**Results:**

During the preweaning period, the relative abundances of propionate producers, including g_*norank_f_Butyricicoccaceae*, g_*Pyramidobacter*, and g_*norank_f_norank_o_Clostridia_vadinBB60_group*, were higher in HADG calves (LDA > 2, *P* < 0.05). Enrichment of these bacteria resulted in increased levels of propionate, a gluconeogenic precursor, in preweaning HADG calves (adjusted *P* < 0.05), which consequently raised serum glucose concentrations (adjusted *P* < 0.05). In contrast, the relative abundances of rumen bacteria in post-weaning HADG calves did not exert this effect. Moreover, no significant differences were observed in rumen fermentation parameters and serum indices between the two groups.

**Conclusions:**

The findings of this study revealed that the preweaning period is the window of opportunity for rumen bacteria to regulate the ADG of calves.

**Supplementary Information:**

The online version contains supplementary material available at 10.1186/s40104-023-00934-0.

## Background

Dairy cows provide high-quality milk to meet the growing demand for nutrients by the population. Therefore, rearing healthy calves for the dairy industry is critical as they form the reserve force for dairy cows. The rearing conditions and growth periods of calves are crucial phases during which any environmental changes can have long-term effects on the growth, health, and milk yield in adult life [1]. A previous study has shown that preweaning liquid feed and solid feed dry matter intake synergistically improve milk, fat, and protein production. In addition, the study also revealed a positive association between first-lactation performance and preweaning ADG [2]. A meta-analysis showed that for every additional 100 g/d increase in preweaning ADG, first-lactation production was expected to increase by 155 kg, and this relationship did not appear to have a plateau [3]. The analysis using a test-day model revealed that the preweaning ADG accounted for 22% of the variation in first lactation milk yield [4]. Together, these studies indicate that an increased preweaning ADG in calves can improve adult lactation performance.

Rumen bacterial composition can affect feed efficiency traits, such as ADG, average daily feed intake, and gain: feed ratio, and has been shown to explain 25.3% of the variation for heifer ADG [5]. Xie et al. [6] suggested that some rumen Lachnospiraceae species involved in carbohydrate metabolism contribute to the variation in residual feed intake. The findings highlighted the potential of the modulation of ruminal microbes or microbial functions to improve the feed efficiency of dairy cows [6]. Xue et al. [7] demonstrated that the rumen microbiota and its metabolites contributed to different milk protein yields. These studies indicated that rumen microbes can influence the host phenotype.

The microbial colonization of the gastrointestinal tract occurs before or during delivery, and methanogens and fibrolytic bacteria have been detected within 20 min after birth [8]. Before weaning or exposure to solid feed, cellulolytic bacterium and other important bacterial species for the proper functioning of the adult rumen were detected on the first day of age [9]. Afterward, the rumen ecosystem undergoes a rapid change by d 3 of age, reflected by an increased abundance of anaerobic genera, a decline in aerobic and facultative anaerobic taxa and an establishment of a new anaerobic environmental niche [9]. The rumen microbes play important roles in the fermentation of the diet and energy supply of the host, which produces volatile fatty acids (VFA) to meet 70% of the daily energy need of a ruminant [10]. Few studies have shown the relationships between ruminal bacteria, VFA and ADG in pre- and post-weaning calves. Therefore, understanding the relationship between ruminal bacteria and ADG in calves could help modulate ruminal bacteria to improve the preweaning ADG of calves.

In this study, we hypothesized that calves with the same dry matter intake (DMI) and diet structure exhibit different rumen fermentation driven by rumen bacteria, leading to variations in energy levels and ultimately affecting the preweaning ADG of the calves. To test this hypothesis, the study aimed to investigate the rumen bacterial composition, rumen fermentation indicators, serum indicators, and growth performance of calves with different ADG. The goal of this study is to provide new insights into calf management.

## Materials and methods

### Animal management, diet and experimental design

This study strictly complied with the requirements of the Animal Ethics Committee of the Chinese Academy of Agricultural Sciences (Beijing, China). Sixty Holstein heifer calves (age, 14 ± 3 d; body weight, 41.25 ± 2.36 kg) were kept in individual hutches till they were 90-day-old. Pasteurized milk (6 L/d) was provided to 14–60-day-old calves (preweaning) twice daily at 0630 and 1700 h. All calves were gradually weaned by reducing the dose of pasteurized milk provided each day by 1 L/d (6, 5, 4, 3, 2, 1, and 1 L/d, respectively) at the age of 61–67 d (weaning period). The claves of 68 to 90-day-old (postweaning) relied solely on solid feed to meet their nutritional needs. All calves had free access to starter feed and clean water throughout the experimental period (Additional file 1: Table S1). Oat hay was fed to all calves at 80 g/d (30–60-day-old) and 200 g/d (61–90-day-old).

The body weight (BW) of 14, 60, 67, and 90-day-old calves was recorded to determine the preweaning, weaning, and postweaning ADG. The initial and left-over amounts of milk, starter feed, and oat hay were recorded to calculate the DMI. The calves were grouped based on their pre- and post-weaning ADG: those with both higher pre- and post-weaning ADG were assigned to the higher ADG group (HADG; *n* = 6), while those with both lower pre- and post-weaning ADG were assigned to the lower ADG group (LADG, *n* = 6). We ensured that the birth weight, DMI and BW of the 14-day-old calves did not differ significantly between the two groups during the grouping process to eliminate the effect of feed and initial weight on ADG. Both groups of calves were descendants of the same bull, aiming to minimize the potential interference of genetic factors.

### Sample collection and measurement

An oral stomach tube was used to collect rumen fluid before morning feeding in 45 and 75-day-old calves [11]. The rumen fluid was collected in two tubes and stored at −80 °C for microbial DNA extraction and VFA concentration determination. The VFA concentration was determined using gas chromatography [12]. Blood samples (10 mL) of each calve (30, 60, and 90-day-old) were collected from the jugular vein before the morning feeding. The blood samples were then centrifuged at 2,000 × *g* for 10 min to collect the serum, which was placed in tubes and frozen at −80 °C for further analyses. The serum parameters, including total protein (TP), albumin (ALB), glucose (GLU), blood urea nitrogen (BUN), glucose (GLU) and immunoglobulins (IgA, IgG, and IgM), were analyzed using a KHB-1280 automatic biochemical analyzer (Kehua Biological Engineering Co., Ltd., Shanghai, China). The levels of globulin (GLB) were also calculated. The concentration of growth hormone (GH), insulin-like growth factor 1 (IGF-1) and insulin (INS) in serum were determined using bovine ELISA kits (Jinhaikeyu Biological Technology Development Co., Ltd., Beijing, China).

### DNA extraction, 16S rRNA gene amplification and sequencing

The microbial DNA was extracted from rumen liquid samples using the E.Z.N.A.® soil DNA Kit (Omega Bio-tek, Norcross, GA, USA) following the manufacturer's instructions. The 1.0% agarose gel electrophoresis and a NanoDrop® ND-2000 spectrophotometer (Thermo Scientific Inc., Waltham, MA, USA) were used to determine the quality and concentration of DNA, respectively. An ABI GeneAmp® 9700 PCR thermocycler (Applied Biosystems, Carlsbad, CA, USA) was used to amplify the hypervariable region V3–V4 of the bacterial 16S rRNA gene with primer pairs 338F (5'-ACTCCTACGGGAGGCAGCAG-3') and 806R (5'-GGACTACHVGGGTWTCTAAT-3') [13]. The PCR reaction mixture comprised 4 μL 5× Fast Pfu buffer, 2 μL 2.5 mmol/L dNTPs, 0.8 μL each primer (5 μmol/L), 0.4 μL Fast Pfu polymerase, 10 ng template DNA, and ddH_2_O to a final volume of 20 µL. The PCR amplification cycling parameters were 95 °C for 3 min, 27 cycles of 95 °C for 30 s, 55 °C for 30 s and 72 °C for 45 s with a final extension at 72 °C for 10 min and end at 4 °C. All samples were amplified in triplicate. The 2% agarose gel was used to extract the PCR product. The PCR product was purified using the AxyPrep DNA Gel Extraction Kit (Axygen Biosciences, Union City, CA, USA) and quantified using Quantus™ Fluorometer (Promega, Madison, WI, USA). Purified amplicons were pooled and paired-end sequenced on an Illumina MiSeq PE300 platform (Illumina, San Diego, CA, USA) at Majorbio Bio-Pharm Technology Co., Ltd. (Shanghai, China).

The raw FASTQ files were demultiplexed using an in-house Perl script. The raw data was quality-filtered using fastp version 0.19.6 [14] and merged using FLASH version 1.2.7 [15] according to the following criteria: (i) the 300 bp reads were truncated at any site receiving an average quality score of < 20 over a 50 bp sliding window, and the truncated reads shorter than 50 bp and reads containing ambiguous characters were discarded; (ii) overlapping sequences longer than 10 bp could be assembled, the maximum mismatch ratio of the overlap region was set at 0.2, and unassembled reads were discarded; (iii) samples were distinguished based on the barcode and primers, and the sequence direction was adjusted, exact barcode matching, 2 nucleotide mismatch in primer matching. Then the optimized sequences were used to cluster operational taxonomic units (OTUs) using UPARSE 7.1 with a 97% sequence similarity level [16]. The RDP Classifier version 2.2 was used to analyze the taxonomy of each OTU representative sequence against the 16S rRNA gene database using a confidence threshold of 0.7 [17]. Metagenomic functions were predicted through the utilization of Phylogenetic Investigation of Communities by Reconstruction of Unobserved States (PICRUSt2), in conjunction with the KEGG (Kyoto Encyclopedia of Genes and Genomes) database [18].

### Statistical analysis

The ADG, DMI, serum parameters, rumen fermentation parameters, and bacterial taxonomic and functional data were analyzed using SAS software (version 9.4, SAS Institute Inc., Cary, NC, USA). A mixed procedure was used as follows:

$${Y}_{ijk}=\mu +{G}_{i}+{P}_{j}({D}_{j})+{GP}_{ij}({GD}_{ij})+{C}_{k}+{e}_{ijk,}$$

where *Y*_*jik*_ is the dependent variable, *μ* is the overall mean, *G*_*i*_ is the effect of the *i*^th^ group, *P*_*j*_ (*D*_*j*_) is the effect of the *j*^th^ period (day), *GP*_*ij*_ (*GD*_*ij*_) is the interaction between group and period (group and day), *C*_*k*_ is the random effect of *k*^th^ calf, and *e*_*ijk*_ is the residual error.

The independent sample *t*-test was performed to analyze birth weight and BW of 14-day-age using the SAS software. The taxonomic and functional data were analyzed on the online platform of the Majorbio Cloud Platform [19]. Rumen microbial data's alpha diversity between groups was assessed using the Wilcoxon rank-sum test, with a false discovery rate (FDR) adjusted *P*-value < 0.05 indicating significance. Principal co-ordinates analysis (PCoA) was applied to assess beta diversity using the Bray–Curtis distance algorithm, while intergroup differences were tested using the adonis method. The taxonomic and functional data were also compared using Linear Discriminant Analysis Effect Size (LEfSe); significance was determined with a *P*-value < 0.05 and a LDA score > 2. The LEfSe was also used to compare the KEGG enzymes with |log_2_ Fold Change| > 1, LDA > 2 and *P*-value < 0.05 considered significant. Spearman's rank correlations were employed to unveil the associations among rumen bacteria at the genus level, with a FDR adjusted *P*-value < 0.05 and a Spearman's |*r*|> 0.50 deemed as statistically significant correlations. The Gephi software (version 0.10) was used for visualization. Spearman’s rank correlations were also constructed between ADG, GLU30 (serum glucose in 30-day-old calves), GLU60 (serum glucose in 60-day-old calves), GH30 (serum growth hormone in 30-day-old calves), GFI-1 30 (serum insulin-like growth factor 1 in 30-day-old calves), INS30 (serum insulin in 30-day-old calves), propionate, total VFA, microorganism and KEGG enzymes during the preweaning period (Spearman's |*r*|> 0.50 and FDR adjusted *P*-value < 0.05). Multiplex networks were visualized using Gephi software (version 0.10).

## Results

### Intake, growth performance and serum parameters

**Fig. 1:**
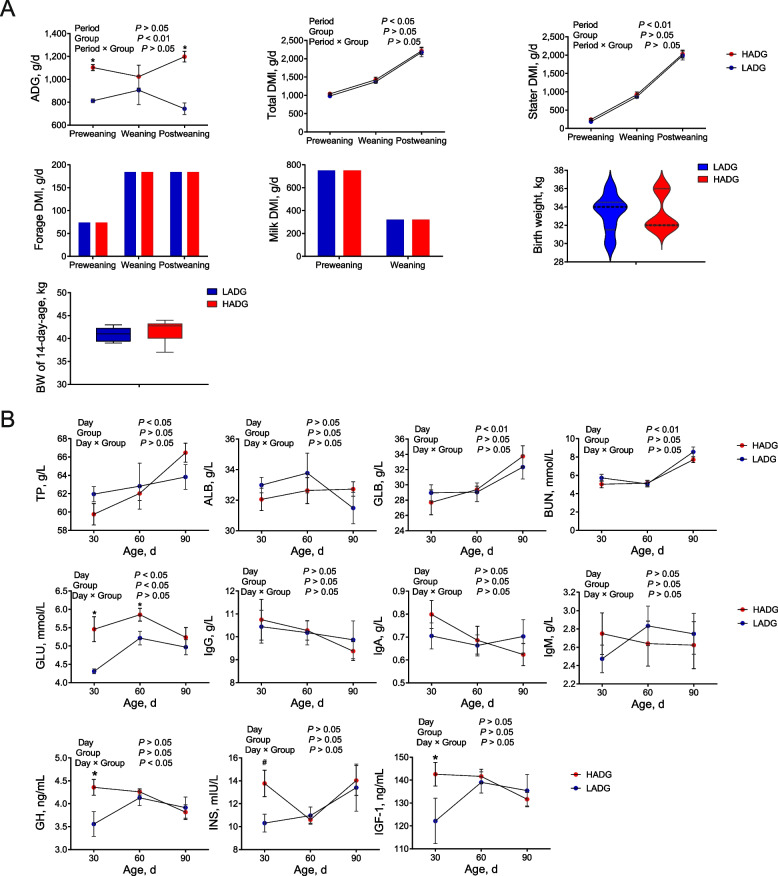
Phenotypes of calves in the HADG and LADG groups at different periods. **A** Differences in feed intake, body weight and ADG between HADG and LADG calves in different periods. **B** Differences in serum indices of HADG and LADG calves in different periods. HADG, higher average daily gain group; LADG, lower average daily gain group; * represents significantly different between the two groups (*P* < 0.05). # represents a tendency to differ between the two groups (0.05 < *P* < 0.10)

The HADG group had significantly higher ADG than the LADG group during the pre- and post-weaning periods (*P* < 0.05) (Fig. 1A). However, the total DMI, stater DMI, birth weight and BW of 14-day-old calves did not differ between the two groups (*P* > 0.05; Fig. 1A). Furthermore, the milk and forage given in a quantified amount and were fully consumed by the calves, which resulted in similar forage DMI, and milk DMI between the two groups during the tested periods (*P* > 0.05; Fig. 1A). These findings suggest that despite consistent birth weight, BW at 14 days of age, total DMI, and diet composition, variations in microbial-mediated ruminal fermentation might cause the differences in ADG.

As shown in Fig. 1B**,** no changes in other serum parameters were observed between the two groups (*P* > 0.05). However, the HADG calves exhibited elevated concentrations of serum GLU30, GLU60, GH30, and IGF-1 30 during the preweaning period compared to those in LADG calves (*P* < 0.05). Additionally, INS30 also showed a tendency to be higher in the serum of HADG calves during the preweaning period (*P* < 0.10). These findings collectively suggest an increase in hormones related to growth and an augmented energy supply in HADG calves (Fig. 1B).

### Ruminal fermentation parameters concerning VFA

**Fig. 2:**
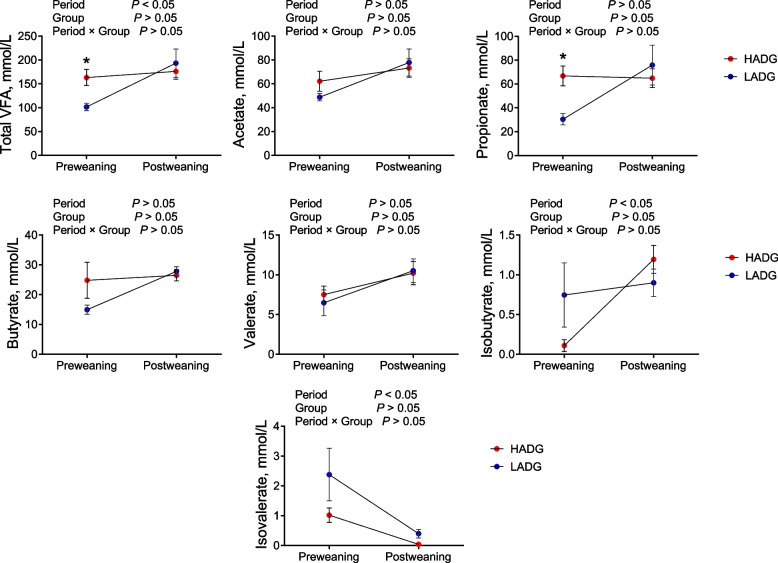
Rumen fermentation in HADG and LADG groups. HADG, higher average daily gain group; LADG, lower average daily gain group; * represents significantly different between the two groups (*P* < 0.05)

Remarkably, higher concentrations of total VFA and propionate were detected in the rumen of HADG calves than those in LADG calves during the preweaning period (*P* < 0.05) (Fig. 2). However, no significant differences in fermentation parameters were observed between the two groups during the postweaning period (*P* > 0.05) (Fig. 2).

### Bacterial diversities and composition

**Fig. 3:**
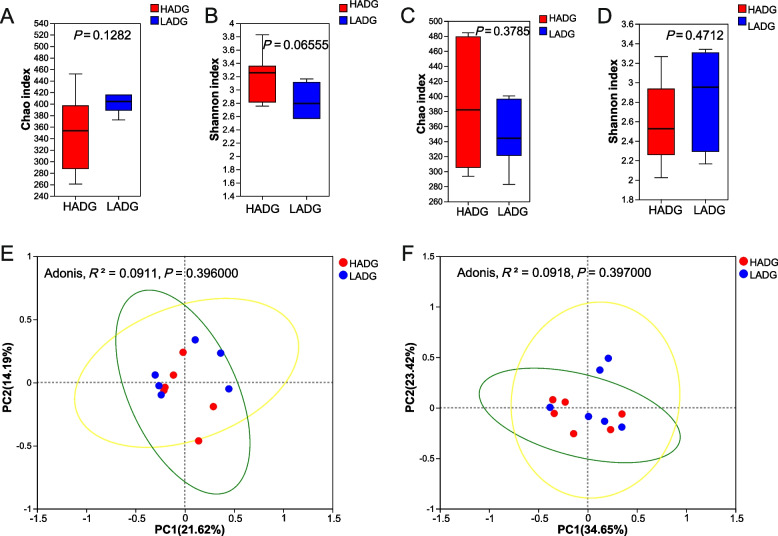
Alpha and beta diversity between HADG and LADG groups in different periods. **A** and **B** Alpha diversity (Chao and Shannon indices) of rumen bacteria between HADG and LADG groups during the preweaning period. **C** and **D** Alpha diversity (Chao and Shannon indices) of rumen bacteria between HADG and LADG groups during the postweaning period. **E** Beta diversity of a principal coordinate analysis (PCoA) based on Bray–Curtis dissimilarity matrices between HADG and LADG groups during the preweaning period. **F** Beta diversity of a principal coordinate analysis (PCoA) based on Bray–Curtis dissimilarity matrices between HADG and LADG groups during the postweaning period. HADG, higher average daily gain group; LADG, lower average daily gain group

Alpha diversity calculations revealed no marked divergence of the Shannon and Chao indices (adjusted *P* > 0.05), indicating unchanged bacterial richness and evenness between the two groups at any stage (Fig. 3A–D). The principal coordinate analysis (PCoA) showed no clear separation between the HADG and LADG groups at any time point (Fig. 3E and F).

**Fig. 4:**
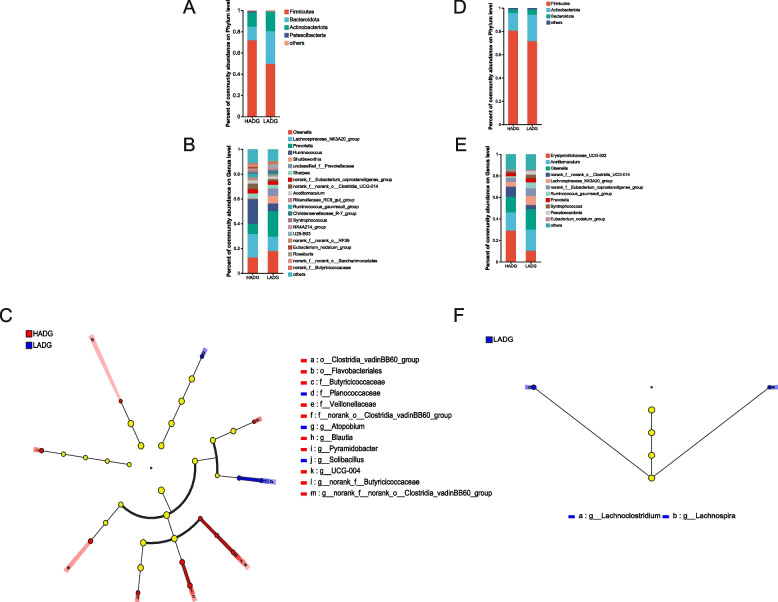
Bacterial composition and differential taxa between HADG and LADG groups in different periods. **A** and **B** The relative abundances of the rumen bacterial community at the phylum and genus levels during the preweaning period. **D** and **E** The relative abundances of the rumen bacterial community at the phylum and genus levels during the postweaning period. **C** and **F** The significantly different bacterial taxa between HADG and LADG groups during the preweaning (**C**) and postweaning period (**F**). HADG, higher average daily gain group; LADG, lower average daily gain group

At the phylum level, the predominant phyla were Firmicutes (61.70%), Bacteroidota (21.42%), Actinobacteriota (15.30%) and Patescibacteria (0.72%) across the 12 samples from the preweaning period (Fig. 4A; Additional file 1: Table S2). Meanwhile, none of the phyla showed significant differences between the HADG and LADG groups. At the genus level, the dominant genera across the 12 samples before weaning were *Lachnospiraceae_NK3A20_group* (15.09%), *Olsenella* (14.82%), *Ruminococcus* (14.18%), *Prevotella* (13.61%) and *Shuttleworthia* (3.38%) (Fig. 4B; Additional file 1: Table S3). Several genera that differed significantly between the two groups were identified; the abundance of most of these genera was higher in the HADG group than that in the LADG group. Specifically, the relative abundances of *norank_f_Butyricicoccaceae*, *Pyramidobacter*, *norank_f_norank_o_Clostridia_vadinBB60_group*, *Blautia*, and *UCG-004* were enriched in the HADG group, whereas the relative abundances of *Solibacillus* and *Atopobium* were enriched in the LADG group (LDA > 2, *P* < 0.05) (Fig. 4C; Additional file 1: Table S4). The support provided by the mixed model further enhances the quality of our results. The results showed that the relative abundances of *norank_f_Butyricicoccaceae* and *norank_f_norank_o_Clostridia_vadinBB60_group* were found to be enriched in the HADG group, while the relative abundances of *Solibacillus* and *Atopobium* were enriched in the LADG group (*P* < 0.05). *Pyramidobacter*, *Blautia*, and *UCG-004* also exhibited a tendency to be higher in the HADG group (*P* < 0.10) (Additional file 2: Fig. S1).

Next, we focused on the samples (*n* = 12) from the postweaning period. The predominant phyla were Firmicutes (77.01%), Actinobacteriota (18.03%), and Bacteroidota (0.72%) (Fig. 4D; Additional file 1: Table S5). No significantly different taxa were found at the phylum level. At the genus level, the dominant genera across the 12 samples from postweaning included *Erysipelotrichaceae_UCG-002* (20.73%), *Acetitomaculum* (17.99%), *Olsenella* (15.75%) and *norank_f__norank_o__Clostridia_UCG-014* (6.77%) (Fig. 4E; Additional file 1: Table S6). However, the abundance of none of these genera differed significantly between the HADG and LADG groups. On the contrary, the abundance of *Lachnospira* and *Lachnoclostridium* was significantly higher in the LADG group than that in the HADG group during the postweaning period (LDA > 2, *P* < 0.05) (Fig. 4F; Additional file 1: Table S7). The mixed model also confirms these results (Additional file 2: Fig. S1).

### Microbial interactions

**Fig. 5:**
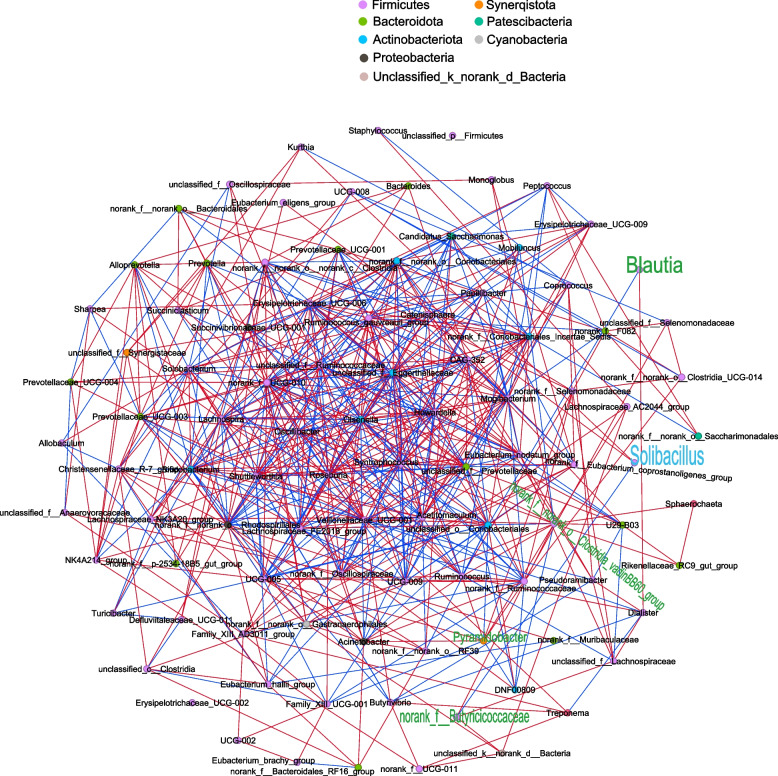
Network analysis to reveal microbial interactions during the preweaning period. The network analysis showed the degree of correlation between the bacteria at the genus level (Spearman's |*r*|> 0.50 and adjusted *P* < 0.05). The node colors represent the phylum classification of the genera. Lines between two nodes represent the correlation, with a red line indicating a positive correlation and a blue line indicating a negative correlation. The green text represents the significantly higher genera in the HADG group; the blue text represents the significantly higher genera in the LADG group (LDA > 2, *P* < 0.05). HADG, higher average daily gain group; LADG, lower average daily gain group

Genera enriched in the rumen of HADG calves had more connections in the network during the preweaning period, suggesting a richer metabolic association and a more critical role in the network. Conversely, the higher abundance of *Solibacillus* in the LADG group during the preweaning period was found to be on the network’s periphery with fewer connections, suggesting a minor role in the overall network (Fig. 5).

During the postweaning period, only *Lachnospira* appeared to be located at the edge of the network. Negative correlations were observed between *Lachnospira* and other taxa (Additional file 2: Fig. S2).

### Functions of microbiota

**Fig. 6:**
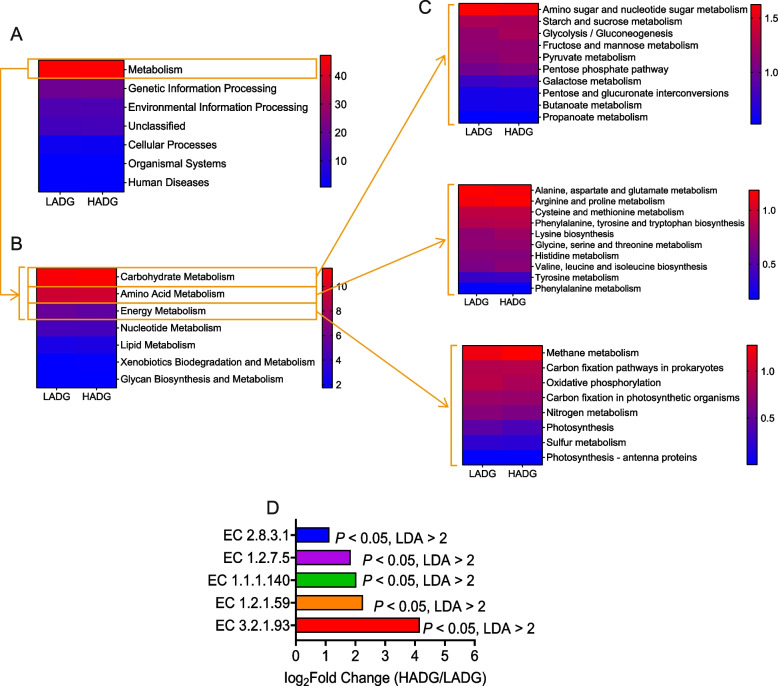
Functional predictions of rumen bacteria between HADG and LADG groups during preweaning period. **A**–**C** Relative abundance of function KEGG pathway level 1, level 2 and level 3 (top 10 of carbohydrate metabolism, top 10 of amino acid metabolism, and top 8 of energy metabolism). **D** Significantly different KEGG enzymes related to carbohydrate and propionate metabolisms between HADG and LADG groups (LDA > 2, *P* < 0.05). HADG, higher average daily gain group; LADG, lower average daily gain group

The software PICRUSt2 was used to predict microbial function. At KEGG pathway level 1, seven categories, including Metabolism (47.07%), Genetic Information Processing (20.65%), Environmental Information Processing (14.72%), Unclassified (13.03%), Cellular Processes (3.00%), Organismal Systems (0.79%), and Human Diseases (0.74%), were identified in the 12 samples during the preweaning period (Fig. 6A). Among these, carbohydrate (11.34%), amino acid (9.57%), and energy (5.47%) metabolism were the most active in the metabolism pathway (Fig. 6B). The top 10 pathways for carbohydrate and amino acid metabolism were further observed at KEGG pathway level 3 (Fig. 6C). However, only eight pathways at KEGG level 3 were detected in the energy metabolism pathway (Fig. 6C). Additionally, several KEGG enzymes related to carbohydrate metabolism and propanoate metabolism were also significantly enriched in preweaning HADG calves (log_2_FC > 1, LDA > 2 and *P* < 0.05) (Fig. 6D; Additional file 1: Table S8). The mixed model can yield identical results as well (*P* < 0.05) (Additional file 2: Fig. S3).

**Fig. 7:**
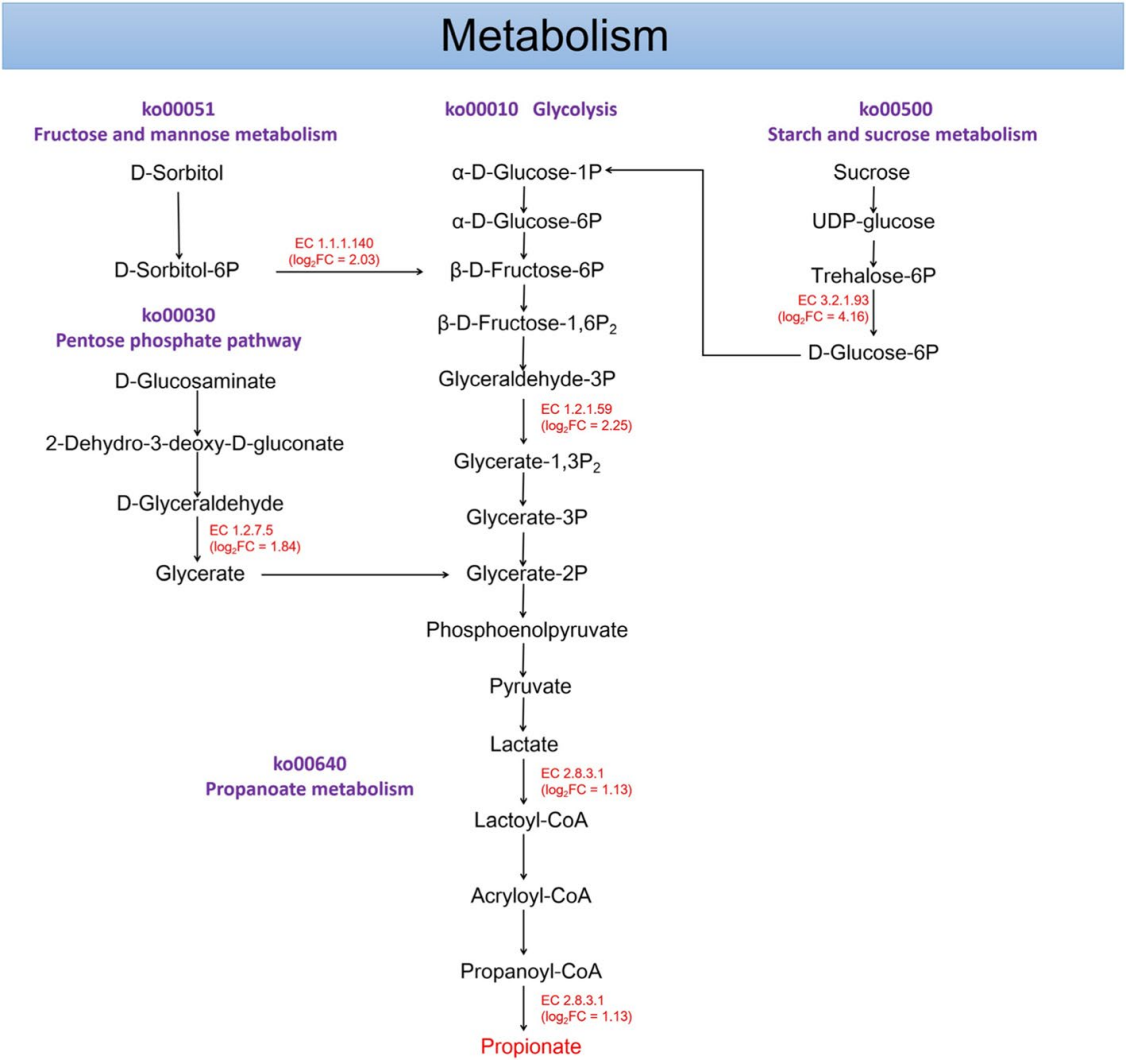
Microbial functions involved in carbohydrate and propionate metabolisms between HADG and LADG groups during the preweaning period. The red text indicates the KEGG enzymes or metabolites enriched in HADG calves. The text in brackets under the KEGG enzyme is HADG/LADG fold change of enzyme. HADG, higher average daily gain group; LADG, lower average daily gain group

The KEGG enzymes related to carbohydrate and propionate metabolisms in the rumen of the preweaning calves were summarized in Fig. 7. Among the KEGG enzymes involved in starch and sucrose metabolism pathway, one enzyme, EC 3.2.1.93, was more abundant in the HADG calves than those in LADG group (log_2_FC > 1, LDA > 2 and *P* < 0.05; Fig. 6D). EC 1.2.1.59 in the glycolysis pathway, EC 1.1.1.140 in the fructose and mannose metabolism pathway, EC 1.2.7.5 in pentose phosphate pathway, and EC 2.8.3.1 in the propanoate metabolism pathway were more abundant in the HADG calves (log_2_FC > 1, LDA > 2 and *P* < 0.05; Fig. 6D).

Next, we focused on the postweaning calves. At the KEGG pathway level 1, 6 categories were observed in the 12 samples during the postweaning period, including Metabolism (76.95%), Genetic Information Processing (8.57%), Environmental Information Processing (6.08%), Cellular Processes (3.79%), Human Diseases (3.00%), and Organismal Systems (1.61%) (Additional file 1: Table S9). In the Metabolism pathway, global and overview maps (41.13%), carbohydrate metabolism (10.81%) and amino acid metabolism (7.09%) were the most active (Additional file 1: Table S10). The top 10 pathways at the KEGG pathway level 3 were involved with carbohydrate and amino acid metabolisms, wherein 8 pathways were identified under the global and overview maps pathway ( Additional file 1: Table S11). None of the KEGG pathways differed significantly. Furthermore, no significant differences were observed in the KEGG enzymes related to carbohydrate and propionate metabolisms. These results suggest that HADG calves lose the advantages imparted by carbohydrate and propionate metabolisms during the postweaning period.

### Relationships between phenotypes, microorganisms, and microbial functions

**Fig. 8:**
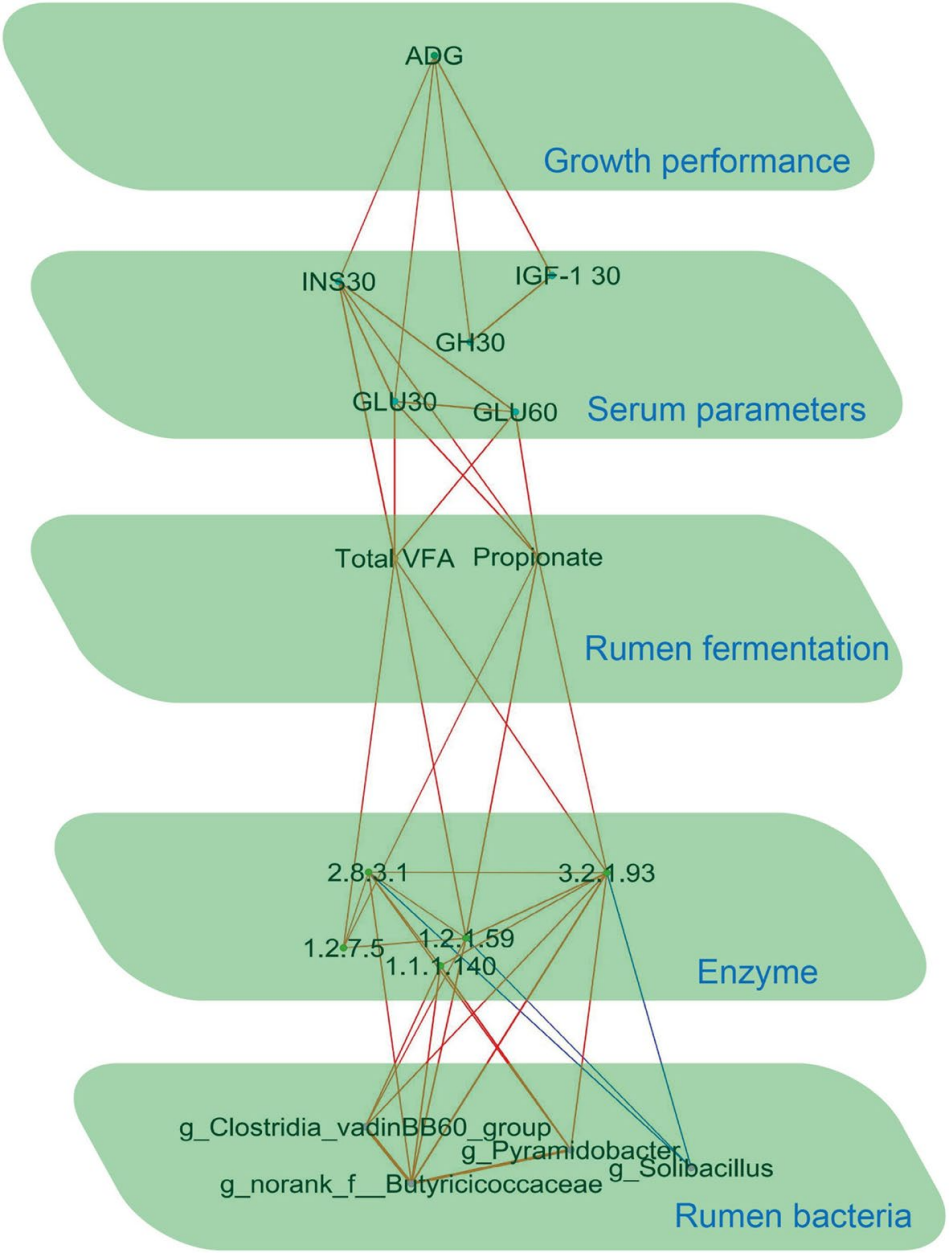
Multiplex networks revealed the relationships between rumen bacteria, KEGG enzymes, rumen fermentation, serum glucose, and host phenotype. Lines between two nodes represent the correlation, with a red line indicating a positive correlation and a blue line indicating a negative correlation (Spearman's |*r*|> 0.50 and adjusted *P* < 0.05)

We investigated the relationships between various indicators by constructing spearman's rank correlations between ADG, GLU30, GLU60, GH30, INS30, IGF-1 30, propionate, total VFA, microorganisms, and KEGG enzymes during the preweaning period. The correlations were visualized by multiplex networks (Fig. 8; Additional file 1: Table S12). *Norank_f_Butyricicoccaceae* positively correlated with the *Pyramidobacter* and *norank_f_norank_o_Clostridia_vadinBB60_group* (adjusted *P* < 0.05). In addition, we identified a few relationships between bacteria and KEGG enzymes. Specifically, the relative abundances *norank_f_Butyricicoccaceae* had positive correlations with the relative abundances of KEGG enzymes related to carbohydrate and propionate metabolisms (EC 1.1.1.140, EC 1.2.1.59, EC 2.8.3.1, and EC 3.2.1.93) (adjusted *P* < 0.05). The relative abundance of *norank_f_norank_o_Clostridia_vadinBB60_group* showed positive correlations with the relative abundances of EC 1.1.1.140, EC 1.2.1.59, and EC 3.2.1.93 (adjusted *P* < 0.05). The *Pyramidobacter* genus showed positive relationships with EC 1.1.1.140, EC 2.8.3.1, and EC 3.2.1.93 (adjusted *P* < 0.05). The concentrations of total VFA and propionate were positively correlated with EC 1.2.1.59, EC 3.2.1.93, and EC 1.2.7.5 (adjusted *P* < 0.05). Propionate is the main precursor for ruminant liver gluconeogenesis, and glucose is the main energy supplier for living organisms. In our study, total VFA and propionate were positively correlated with INS30, GLU30 and GLU60 (adjusted *P* < 0.05), wherein serum GLU30 and INS30 showed a positive correlation with ADG (adjusted *P* < 0.05).

## Discussion

In this study, we systematically compare ruminal bacteria and host phenotypes of calves with different ADG in pre- and post-weaning periods. Our study sheds light on the role of ruminal bacteria in driving variation in preweaning ADG. Our data indicate that rumen bacteria play a pivotal role in propionate and total VFA production, leading to elevated serum glucose and insulin levels, ultimately contributing to a higher preweaning ADG.

The differences in total DMI were likely responsible for most of the improvement in preweaning ADG, as the higher DMI can provide the necessary energy and protein needed for growth [20]. Additionally, diet structure can also affect the growth performance of calves, with liquid feeds being shown to meet the energy needs of calves better than solid feeds [21]. A previous study has also shown that increased milk intake positively affects calf growth during preweaning [22]. However, in our study, we did not observe any differences in total DMI or diet structure between the two groups, suggesting other factors are responsible for the difference in preweaning ADG.

Further analysis of serum indicators revealed that serum GLU, INS, GH, and IGF-1 were higher in HADG calves than that in LADG calves during the preweaning period, and the these differences between the two groups disappeared during the postweaning period. Glucose is an essential energy-producing nutrient for all mammals and is important for the survival and growth of cattle [23]. Blood glucose concentration in ruminants is closely related to the glucose turnover rate, meaning that a high blood glucose level can lead to increased glucose utilization [24]. Insulin is an important metabolic hormone that regulates glucose concentration and stimulates the uptake and utilisation of glucose by tissues [25]. Growth hormone and its closely associated hormone, insulin-like growth factor 1, play a pivotal role in stimulating bodily growth and advancement by exerting their effects on vital metabolic organs such as the liver, skeletal muscle, and bones [26]. Hence, elevated levels of glucose, growth hormone, insulin, and insulin-like growth factor 1 in serum potentially supply greater energy to calves, enhancing glucose utilization and potentially fostering bodily growth and development. This may ultimately contribute to variations in preweaning ADG.

The reasons for the differences in serum parameters still need to be further explored. In terms of rumen fermentation indicators, we found important clues. During the preweaning period, the HADG calves had higher total VFA and propionate concentrations. Approximately 70% of the host’s energy needs can be met by VFA energy produced by rumen microbes [27]. Cattle primarily obtain glucose from ruminal VFAs through gluconeogenesis [28]. Undoubtedly, higher total VFA concentrations are beneficial for increasing host energy levels and promoting host growth and development. Propionate, valerate and isobutyrate can be used as precursors of hepatic glucose production for the net synthesis of glucose and as the main source of glucose and energy for ruminants [27, 29, 30]. Propionate is the main precursor substance for hepatic gluconeogenesis, with a contribution rate of 60%–74% [28]. In this study, the accumulation of propionate in the rumen of HADG calves during the preweaning period resulted in elevated serum glucose.

Ruminal bacteria are responsible for driving ruminal fermentation, which produces VFAs [31]. Hence, analyzing rumen bacteria is necessary to investigate the reasons for changes in VFAs. Similar to previous studies, Firmicutes and Bacteroidota were the two major bacterial phyla detected in preweaning calves [32, 33]. Firmicutes have been shown to be dominant in high-grain diets, while the Bacteroidota are dominant in hay diets [34]. In this study, preweaning calves were primarily fed starter and less hay, which may have led to the dominance of the Firmicutes phylum. Almost all differential genera (*norank_f_Butyricicoccaceae*, *Pyramidobacter*, *norank_f_norank_o_Clostridia_vadinBB60_group*, *Blautia*, and *UCG-004*) had higher abundance in the rumen of HADG calves during the preweaning period.

The Butyricicoccaceae family is an important butyrate producer [35]. Our study demonstrated that *norank_f_Butyricicoccaceae* likely possess an acid-producing function, leading to differences in rumen fermentation. *Pyramidobacter* contains many acid-producing strains that produce acetate, isovalerate, propionate, isobutyrate, succinate, and phenylacetic acid as end products [36, 37]. A Previous study revealed that *Pyramidobacter* may synthesize thiamine (vitamin B1) in the rumen to meet the needs of ruminants [38]. Additionally, the *Pyramidobacter* genus was found to be associated with rumen development [39]. In short, the *Pyramidobacter* genus not only can produce acid but also may produce other substances beneficial to the host, which may promote the growth and development of the host.

The Clostridiales vadinBB60 group is known to consist of conditional pathogens [40]. Under normal conditions, a balance is maintained between conditional pathogens and their hosts and other flora through factors such as nutritional competition and mutual restraint of metabolites. However, this equilibrium relationship is disrupted under certain conditions, and the conditional pathogens may show a negative impact. This suggests that conditional pathogens may also perform beneficial functions. Zhang et al. [41] suggested that the unclassified Clostridiales vadinBB60 group was positively correlated with propionate. In this study, the enrichment of *norank_f_norank_o_Clostridia_vadinBB60_group* may also be a reason for the increased propionate concentration.

*Blautia* is a genus of probiotic bacteria widely distributed in mammalian feces and intestines and expresses enzymes related to propionate production [42, 43]. *Blautia* is also positively related to plasma concentrations of amino acids, biogenic amines, and sphingomyelins, as well as ADG in calves [44]. In terms of microbial networks, *norank_f_Butyricicoccaceae*, *Pyramidobacter*, *norank_f_norank_o_Clostridia_vadinBB60_group*, and *Blautia* have more connections with other bacterial taxa, especially *Pyramidobacter,* occupies a relatively important position in the network. Enrichment of these four genera in the rumen of HADG calves may have increased the metabolic efficiency of the rumen flora [45], resulting in a greater impact on the rumen flora.

The relative abundance of *Solibacillus* was increased in the LADG calves during the preweaning period. A higher predisposition to diarrhea may be associated with the *Solibacillus* [46]. This indicates that the enrichment of the *Solibacillus* is detrimental to the growth and development of the host. Nevertheless, the network analysis identified *Solibacillu*s at the edge of the microbial network indicating its less connection to other taxa and less influence on the network.

During the postweaning period, Firmicutes and Actinobacteria emerged as the dominant bacterial phyla. On the contrary, previous studies have reported Bacteroidetes and Firmicutes as the main bacterial phylum in postweaning calves [47]. This discrepancy could be attributed to the different dietary structures in the studies, which may have contributed to the enrichment of Actinobacteria. All the differential genera (*Lachnospira* and *Lachnoclostridium*) were enriched in postweaning LADG calves, suggesting that the HADG calves have lost the advantage of acidogenic bacteria, as evidenced by ruminal fermentation parameters during the postweaning period. *Lachnospira* comprises many pectin degradation bacteria and has been shown to be enriched in the rumen of grass-fed steers [48, 49]. *Lachnoclostridium* consists of many beneficial bacteria [50]. However, an increase in the relative abundances of *Lachnospira* and *Lachnoclostridium* was insufficient to alter ruminal fermentation in LADG calves.

The KEGG enzymes predicted by PICRUSt2 provide a way to evaluate the functions of rumen microorganisms. During the preweaning period, five enzymes (EC 2.8.3.1, EC 1.2.7.5, EC 1.1.1.140, EC 1.2.1.59, and EC 3.2.1.93) related to carbohydrate and propionate metabolisms were enriched in the HADG calves. Propionyl-CoA carboxylase (EC 2.8.3.1), a key enzyme, played a vital role in propionate formation via propanoate metabolism [51]. Aldehyde ferredoxin oxidoreductase (EC 1.2.7.5) is involved in the pentose phosphate pathway, and its catalyzed downstream metabolites are involved in the Glycolysis. Sorbitol-6-phosphate 2-dehydrogenases (EC 1.1.1.140) catalyze the interconversion of *D*-sorbitol-6-phosphate to *D*-fructose 6-phosphate, which can be utilized by bacteria to utilize sorbitol [52]. Glyceraldehyde-3-phosphate dehydrogenase (EC 1.2.1.59) promote the degradation of carbohydrates in the glycolysis pathway [53]. Phosphotrehalase (EC 3.2.1.93) promotes sucrose degradation in the starch and sucrose metabolism. Overall, the enrichment of the five KEGG enzymes may promote the degradation of carbohydrates in the fructose and mannose metabolism, starch and sucrose metabolism, pentose phosphate pathway, and glycolysis pathway. They also support propanoate metabolism by synthesizing lactate from pyruvate, resulting in an increased propionate concentration. However, during the postweaning period, the two groups of calves did not differ significantly in KEGG enzymes involved in carbohydrate degradation and propionate metabolism.

Multiplex networks revealed that rumen microbes regulate ADG during the preweaning period. The abundance of three genera (*norank_f_Butyricicoccaceae*, *Pyramidobacter*, and *norank_f_norank_o_Clostridia_vadinBB60_group*) led to the enrichment of three KEGG enzymes (EC 1.2.1.59, EC 1.2.7.5, and EC 3.2.1.93) related to carbohydrate degradation, which in turn increased the concentrations of propionate and total VFA. Propionate can be used as a substrate for hepatic gluconeogenesis to produce glucose and promote serum glucose concentrations. Higher serum glucose concentrations not only provide calves with more energy but also increase the efficiency of serum glucose utilization in calves, ultimately resulting in higher ADG. Concurrently, heightened serum glucose levels prompt the body to generate insulin, thereby further amplifying glucose utilization and providing the body with increased energy resources.

It is important to emphasize that the HADG calves maintained higher ADG even during the postweaning period, but this was not due to differences in rumen fermentation driven by microorganisms. In other words, the window of microbial regulation of ADG may disappear during the postweaning period, but the advantage of higher ADG caused by microorganisms during the preweaning period will persist for a long time.

## Conclusion

**Fig. 9:**
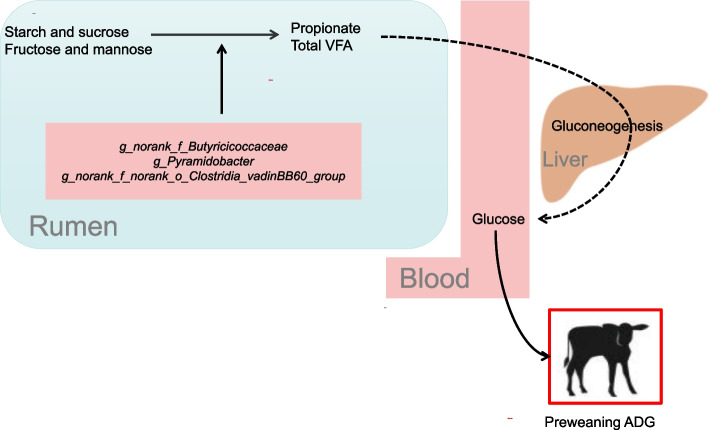
The preweaning period is the window of opportunity for rumen bacteria to regulate the average daily gain (ADG). VFA, volatile fatty acids

In conclusion, this study highlights the preweaning period as a critical time window for rumen bacteria to regulate ADG (Fig. 9). Specifically, the abundance of three key genera (*norank_f_Butyricicoccaceae*, *Pyramidobacter*, and *norank_f_norank_o_Clostridia_vadinBB60_group*) with propionate-producing and carbohydrate-degrading abilities in preweaning calves led to higher concentrations of propionate and total VFA. The bacteria-driven production of propionate and total VFA were strongly correlated with preweaning serum glucose and insulin, which in turn were positively associated with preweaning ADG. These correlation networks are crucial in regulating multiple host phenotypes during preweaning, particularly preweaning ADG. It is important to note that although the window period for rumen bacteria to regulate ADG disappears after weaning, the beneficial effects of bacteria on ADG can persist in the postweaning period. Further studies are needed to confirm these findings and to explore the potential long-term effects of these differences on the health and productivity of the animals.

### Supplementary Information

**Additional file 1: Table S1.** The composition and nutrient levels of starter diets (DM basis), %. **Table S2.** The relative abundances of the top 10 phyla between HADG and LADG groups during the preweaning period. **Table S3.** The relative abundances of the top 10 genera between HADG and LADG groups during the preweaning period. **Table S4.** The relative abundances of significantly different genera between HADG and LADG groups during the preweaning period. **Table S5. **The relative abundances of the top 9 phyla between HADG and LADG groups during the postweaning period. **Table S6.** The relative abundances of the top 10 genera between HADG and LADG groups during the postweaning period. **Table S7.** The relative abundances of significantly different genera between HADG and LADG groups during the postweaning period. **Table S8.** The relative abundances of significantly different KEGG enzymes between HADG and LADG groups during the preweaning period. **Table S9.** The relative abundances of KEGG pathways level 1 between HADG and LADG groups during the postweaning period. **Table S10.** The relative abundances of KEGG pathways level 2 between HADG and LADG groups during the postweaning period. **Table S11.** The relative abundances of KEGG pathways level 3 between HADG and LADG groups during the postweaning period. **Table S12.** Correlation analysis to reveal interactions among the significantly different bacterial genera, enzymes, fermentation parameters, serum indicators and ADG during the preweaning period.

**Additional file 2: Fig. S1.** The mixed model reveals significantly different bacterial genera. **Fig. S2.** Network analysis to reveal microbial interactions during the postweaning period. **Fig. S3.** The mixed model reveals significantly different KEGG enzymes.

## Data Availability

All data involved in this study are included in this article and its Supplementary files.

## Abbreviations

ADG: Average daily gain
ALB: Albumin
BUN: Blood urea nitrogen
DMI: Dry matter intake
GH30: Serum growth hormone in 30-day-old calves
GLB: Globulin
GLU30: Serum glucose in 30-day-old calves
GLU60: Serum glucose in 60-day-old calves
HADG: Higher average daily gain
IGF-1 30: Serum insulin-like growth factor 1 in 30-day-old calves
INS30: Serum insulin in 30-day-old calves
KEGG: Kyoto Encyclopedia of Genes and Genomes
LADG: Lower average daily gain
TP: Total protein
VFA: Volatile fatty acids
