# Supplementary material for: Preweaning period is a critical window for rumen microbial regulation of average daily gain in Holstein heifer calves
Source: J Anim Sci Biotechnol. 2023 Nov 2;14:128. doi: 10.1186/s40104-023-00934-0 (PMC10621147; doi:10.1186/s40104-023-00934-0)
Supplement: Supplementary file 2 — Additional file 2: Fig. S1. The mixed model reveals significantly different bacterial genera. Fig. S2. Network analysis to reveal microbial interactions during the postweaning period. Fig. S3. The mixed model reveals significantly different KEGG enzymes. [file 40104_2023_934_MOESM2_ESM.docx]

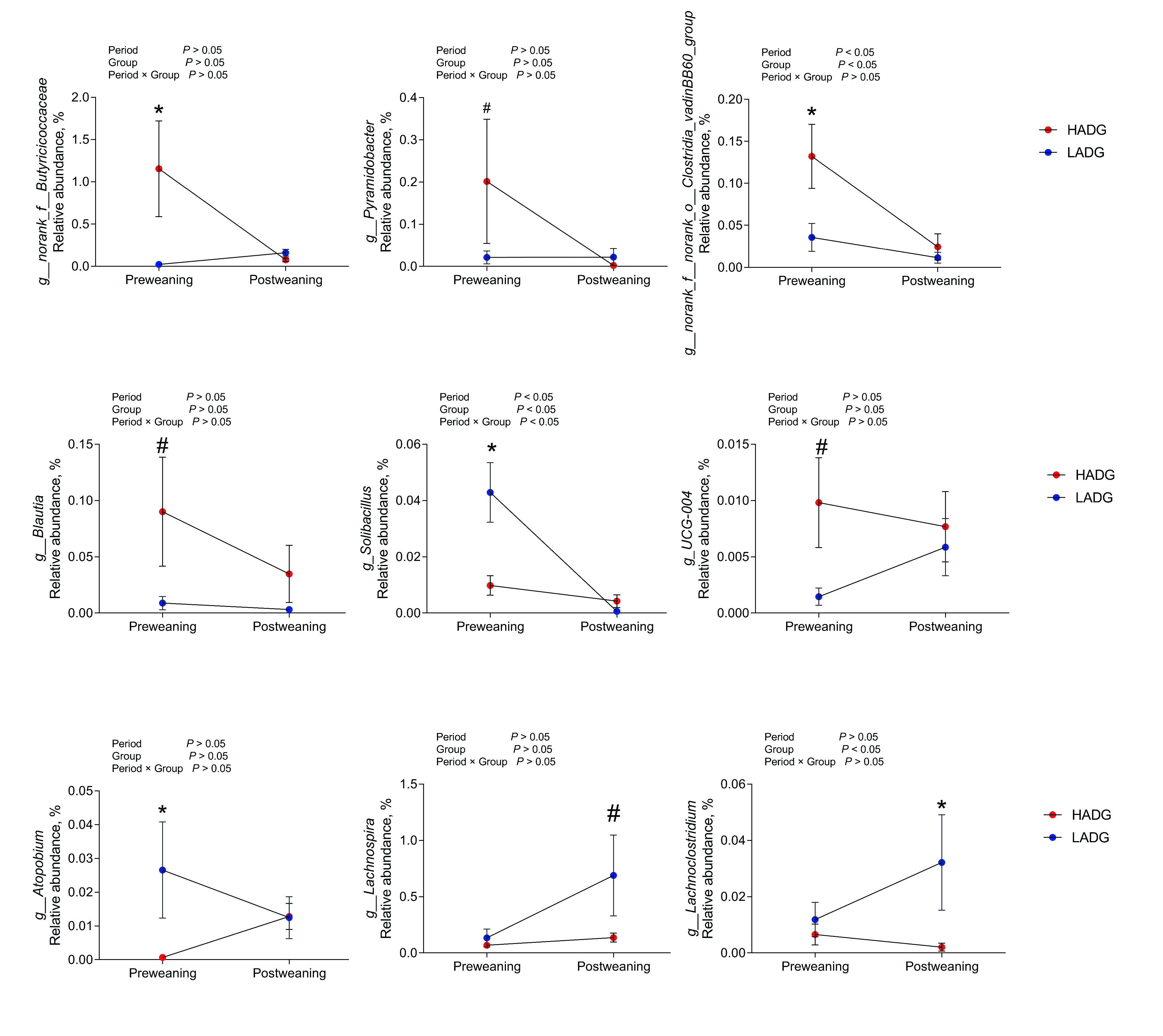


**Fig. S1** The mixed model reveals significantly different bacterial genera. * represents significantly different between the two groups (*P* < 0.05). # represents a tendency to differ between the two groups (0.05 < *P* < 0.10)


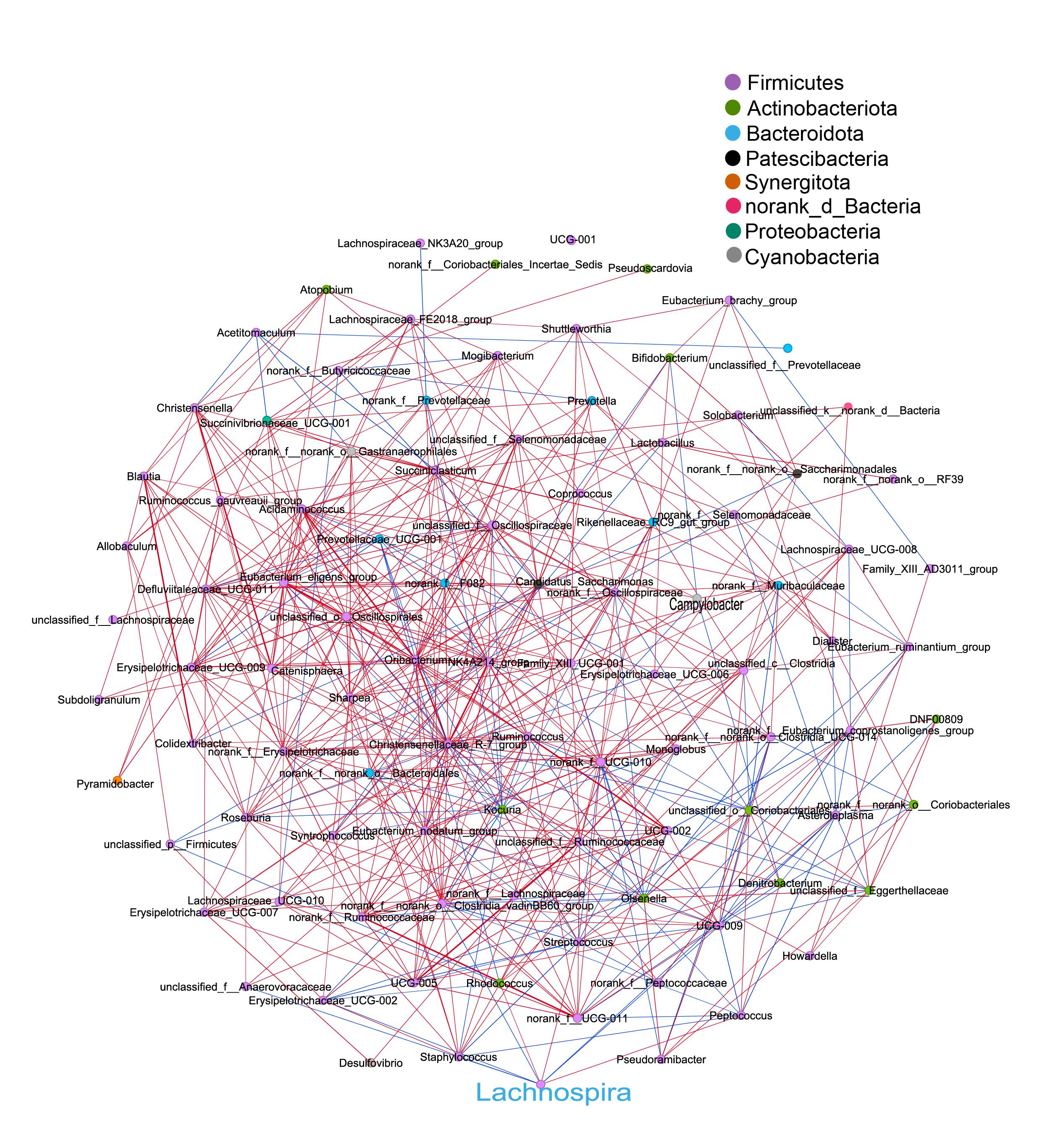


**Fig. S2** Network analysis to reveal microbial interactions during the postweaning period. The network analysis showed the degree of correlation between the bacteria at the genus level (Spearman’s |*r*| > 0.50 and adjusted *P* < 0.05). The node colors represent the phylum classification of the genera. Lines between two nodes represent the correlation, with a red line indicating a positive correlation and a blue line indicating a negative correlation. The green text represents the significantly higher genera in the HADG group; the blue text represents the significantly higher genera in the LADG group (LDA > 2, *P* < 0.05). HADG, higher average daily gain group; LADG, lower average daily gain group

.


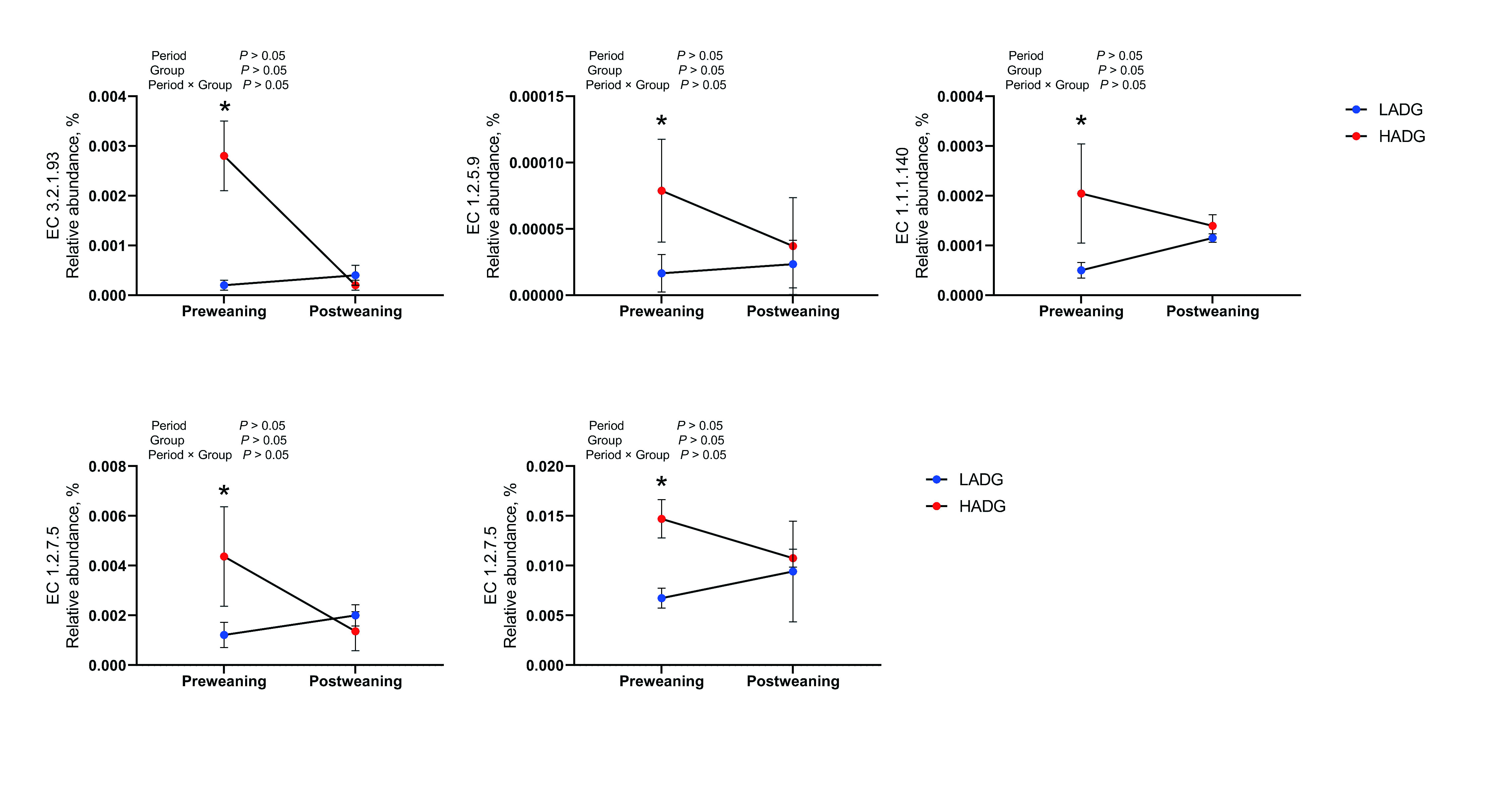


**Fig. S3** The mixed model reveals significantly different KEGG enzymes. * represents significantly different between the two groups (*P* < 0.05)
